# Recovering metals from aqueous solutions by biosorption onto phosphorylated dry baker’s yeast

**DOI:** 10.1038/s41598-018-36306-2

**Published:** 2019-01-18

**Authors:** Yoshihiro Ojima, Shogo Kosako, Maya Kihara, Norikazu Miyoshi, Koichi Igarashi, Masayuki Azuma

**Affiliations:** Department of Applied Chemistry and Bioengineering, Osaka City University, 3-3-138, Sugimoto, Sumiyoshi-ku, Osaka 558-8585 Japan

## Abstract

Biosorption is a cost-effective and simple technique for removing heavy metals and rare earth elements from aqueous solution. Here, metals were recovered from aqueous solutions using phosphorylated dry baker’s yeast cells. The cells were phosphorylated using cyclo-triphosphate, Na_3_P_3_O_9_. The total P content of the phosphorylated cells was ~1.0 mmol/g dry cell weight (DCW). The zeta potential of the phosphorylated cells was −45 mV, two times higher than for the non-phosphorylated cells. The strong negative charges of the phosphorylated cells allowed the cells to adsorb heavy metal ions such as Cd^2+^, Cu^2+^, Pb^2+^, and Zn^2+^, the adsorption capacities of which reached ~1.0 mmol/g DCW. This adsorption capacity was the highest level found in the previous studies using yeast dead biomass. The adsorbed metal ions were easily desorbed in 0.1 M HCl. The phosphorylated cells also adsorbed rare earth ions including Ce^3+^, Dy^3+^, Gd^3+^, La^3+^, Nd^3+^, Y^3+^, and Yb^3+^ with high efficiency. Furthermore, the phosphorylated yeast cells selectively adsorbed the rare earth ions (Nd^3+^ and Yb^3+^) from a solution containing heavy metals and rare earth ions because trivalent positively charged ions were adsorbed preferentially over divalent ions. Thus, phosphorylated yeast cells therefore have great potential for use as novel bioadsorbents. It is also expected that this technique can be applied to many microbial materials as well as yeast.

## Introduction

Biosorption is a cost-effective and simple technique for removing heavy metals or rare earth elements from effluent. Biosorption relies on the ability of living and/or non-living biomass to rapidly adsorb and concentrate (through physicochemical pathways) heavy metal or rare earth ions from even dilute aqueous solutions^1–3^.

Many biomaterials (e.g., algae, bacteria, by-products of animal origin, food industry and agricultural waste, fungi, plants, and yeasts) have been used to biosorb metals^2–10^. *Saccharomyces cerevisiae* has received a great deal of attention because of its unique characteristics. *S. cerevisiae* in various forms (e.g., food industry waste, immobilised yeast, commercial baker’s yeast, laboratory-cultivated baker’s yeast, other laboratory-cultivated yeasts, and magnetically, chemically or thermally modified yeasts) can remove toxic metals (e.g., Cd, Hg, Pb, and Zn), radionuclides (e.g., Ce, Cs, Sr, and U), precious metals (e.g., Ag, Au, Pd, and Pt), and light metals (e.g., Al) from aqueous solutions^11^. Even now, yeast cells have been intensively studied from the aspect of increasing the adsorbent capacity and new application for biomineralization^12–16^. Yeast cells can be obtained as a by-product of the fermentation industry, and are therefore an accessible form of biomass for use as a bioadsorbent^11,12,17^.

The biosorption of metals is a complex process that is affected by the adsorbent, the types and concentrations of metals in the solution to be treated, and other environmental factors. Developments in molecular biology have made it possible to use molecular tools to engineer living microorganisms. Valuable microbial functions have been biochemically and genetically characterised, and microorganisms have been engineered to perform these functions^13,18^. In the microbial cell surface display technique, a heterologous protein/peptide of interest is expressed fused with various cell-surface proteins or fragments^1^. Using this technique, a target metal-binding protein/peptide can be expressed and displayed on the cell surfaces fused with an anchor protein to enhance the metal adsorption^15–18^. Although these approaches remarkably improved the metal adsorption of yeast cells, adsorption capacities are still much lower than those of inorganic adsorbent including ion-exchange resin (0.6~3 mmol/g). Furthermore, these bioadsorbents should be used under mild condition to avoid the damage of displayed protein/peptide or cell death.

In contrast, non-living microbial biomass offers advantages over living microorganisms when biosorption is performed. Metal adsorption is possible not only on the cell surface but also inside the cells as there are no penetration barriers associated with the cell membrane. Non-living microbes do not require nutrients and are not affected by toxic heavy metals. In addition, non-living biomass can be stored for long periods^19,20^. Physical and chemical biomass pretreatment methods can improve the adsorption qualities of the biomass^14^. Among them, phosphorylated biomass is expected to be an excellent bioadsorbent of cationic metal ions because of the strong negative charges on the phosphate groups^21^. In particular, phosphorylating using inorganic sodium cyclo-triphosphate, Na_3_P_3_O_9_ (P_3m_), is a safe and efficient technique and phosphorylated cellulose has been used to adsorb metal ions^22^. It is also well summarized that P_3m_ is a very useful agent for phosphorylating alcohols, amines, amino acids, and sugars in aqueous solutions^23^. From this point of view, phosphorylation of non-living microbial biomass is a promising method to develop a novel biosorbent because such biomass is complex, and constructed by organic substances such as amines, amino acids, and sugars.

In this study, dry baker’s yeast cells were phosphorylated using P_3m_. The phosphorylation efficiency and surface electric charges on the non-living phosphorylated yeast cells were determined. The phosphorylated yeast cells were then used in metal adsorption experiments. The amounts of heavy metal and rare earth ions adsorbed by the phosphorylated yeast cells were determined. Furthermore, desorption of copper ions adsorbed to the phosphorylated yeast cells was examined. Finally, the selective adsorption of rare earth ions from a mixture of ions was performed using the phosphorylated yeast cells. This is the first report endowing the yeast cells with negative charge by installing the anionic functional group for biosorption.

## Results and Discussion

### Phosphorylation of yeast cells and the properties of the phosphorylated cells

**Figure 1:**
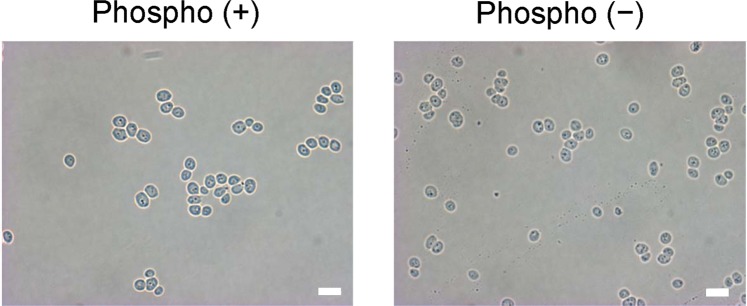
Microscopy images of non-phosphorylated yeast cells (phospho (−) cells, used as negative controls) and yeast cells that had been phosphorylated using cyclo-triphosphate, Na_3_P_3_O_9_, called phospho (+) cells (each scale bar indicates 10 µm).

Yeast cells were phosphorylated using P_3m_ following a method previously used to phosphorylate cellulose^22^. Microscopy images of the phospho (+) and phospho (−) cells are shown in Fig. 1. The sizes or shapes of phospho (+) and phospho (−) cells were not markedly different. The yeast cells clearly retained their normal shapes when they were phosphorylated, suggesting that phosphorylation did not destroy the cell structures.

The degree to which the yeast cells were phosphorylated by P_3m_ was evaluated by performing elemental analysis using the vanadomolybdate method. The P content was normalised to the DCW of the yeast cells. As shown in Table 1, the P content of the phospho (−) cells produced in the first test was 0.07 mmol/g DCW, and the P content of the phospho (+) cells was approximately 10 times higher, 0.91 mmol/g DCW. The P contents of the phospho (−) and phospho (+) cells produced in the second test were 0.06 and 1.09 mmol/g DCW, respectively. The elemental analysis results indicate that the yeast cells were successfully phosphorylated using P_3m_ and that the phosphorylation process was reproducible.

**Table 1:** P contents (determined by elemental analysis) and zeta potentials of the phospho (+) and phospho (−) yeast cells.

	P content (first test) [mmol/g DCW]	P content (second test) [mmol/g DCW]	Zeta potential [mV]
Phospho (+) cells	0.91	1.09	−45.2 ± 4.7
Phospho (−) cells	0.07	0.06	−26.3 ± 7.3

Adding PO_4_^2^^−^ groups may increase the negative charges on yeast cells; therefore, the zeta potentials of rehydrated phospho (+) and phospho (−) cells were compared. The zeta potential of the phospho (−) cells was  −26 mV (Table 1), which was comparable to the zeta potentials of normal yeast cells found in a previous study^24^. The zeta potential of the phospho (+) cells was −45 mV, approximately two times higher than the zeta potential of the phospho (−) cells. We therefore concluded that the phospho (+) cells were markedly more negatively charged than the non-phosphorylated cells. The increase in the zeta potential of the cells through phosphorylation supported our conclusion that the yeast cells were successfully phosphorylated.

### Adsorption of heavy metal ions to phosphorylated yeast cells

**Figure 2:**
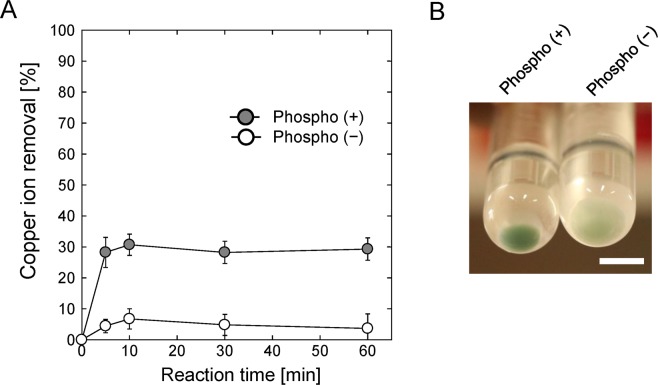
Cu^2+^ adsorption onto phospho (+) and phospho (−) yeast cells. (**A**) Time course for Cu^2+^ removal. Cu^2+^ concentration was 100 ppm and the yeast cell concentration was 0.5 mg/mL. (**B**) Photographs of the phospho (+) and phospho (−) yeast cells after they had been used to adsorb Cu^2+^. The scale bar indicates 10 mm.

It has previously been found that phosphorylated cellulose paper adsorbed appreciable amounts of metal ions, which became bound to the PO_4_^2−^ groups^24^. We investigated the adsorption of metal ions to phosphorylated yeast cells. First, Cu^2+^ adsorption time courses for phospho (+) and phospho (−) cells were compared (Fig. 2). These experiments were performed using a Cu^2+^ concentration of 100 ppm and a yeast cell concentration of 0.5 mg/mL. The phospho (−) cells removed 4% of the Cu^2+^ ions in 5 min and ~5% in 10 min, but did not remove more as the time increased further. The phospho (+) cells removed ~28% of the Cu^2+^ ions in 5 min and ~30% in 10 min, but did not remove more as the time increased further (~29% of the Cu^2+^ ions had been removed in 60 min). These results indicated that the adsorption rate was very high and that adsorption equilibrium was reached within 10 min. An adsorption period of 10 min was therefore used in subsequent experiments. Photographs of the precipitated phospho (+) and phospho (−) cells after they had been used to adsorb Cu^2+^ ions are shown in Fig. 2B. The phospho (+) cells were much more visibly blue than the phospho (−) cells because more Cu^2+^ was adsorbed to the phospho (+) cells than to the phospho (−) cells.

**Figure 3:**
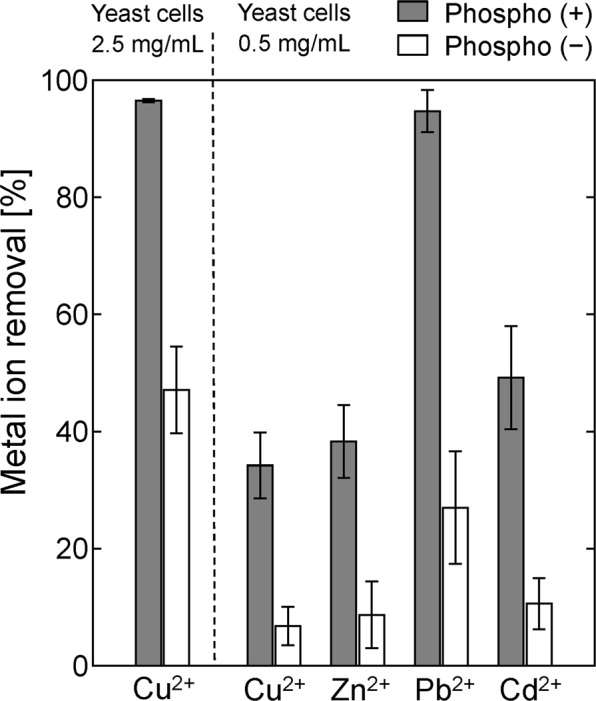
Adsorption of heavy metal ions onto the phospho (+) and phospho (−) yeast cells. The percentages of Cd^2+^, Cu^2+^, Pb^2+^, and Zn^2+^ ions removed in 10 min. The metal ion concentration was 100 ppm (Cd^2+^ 0.89 mM, Cu^2+^ 1.57 mM, Pb^2+^ 0.48 mM, and Zn^2+^ 1.53 mM), and the yeast cell concentration was 0.5 or 2.5 mg/mL.

The percentages of various heavy metal ions adsorbed by the phospho (+) and phospho (−) cells are shown in Fig. 3. In experiments using yeast cells at a concentration of 0.5 mg/mL, the phospho (+) cells and phospho (−) cells removed ~34% and ~7%, respectively, of the Cu^2+^ ions. Much larger proportions of the metal ions were adsorbed by the phospho (+) cells than by the phospho (−) cells. In experiments using yeast cells at a concentration of 2.5 mg/mL, the phospho (+) cells removed 97% of the Cu^2+^ ions. These results suggest that phosphorylated yeast cells are excellent bioadsorbents of metal ions. Cd^2+^, Pb^2+^, and Zn^2+^ were also adsorbed more efficiently by the phospho (+) cells than by the phospho (−) cells at a cell concentration of 0.5 mg/mL. The phospho (−) cells removed ~27% of the Pb^2+^ ions. This was because the molar concentration of Pb^2+^ ions was relatively low because the same mass concentration (100 ppm) was used as was used for the other metal ions even though the atomic weight of Pb (207.2) is much higher than the atomic weights of the other metals.

The adsorption capacities of the phospho (+) and phospho (−) cells for the different metal ions that were used are summarised in Table 2. The Cu^2+^ adsorption capacity of the phospho (−) cells was 0.21 ± 0.10 mmol/g DCW, comparable to the adsorption capacity of dead yeast cells found in a previous study^25^. The Cu^2+^ adsorption capacity of the phospho (+) cells was 1.08 ± 0.18 mmol/g DCW, markedly higher than the adsorption capacities of the phospho (−) cells. The Cd^2+^, Pb^2+^, and Zn^2+^ adsorption capacities of the phospho (+) cells were all ~1.0 mmol/g DCW. The high adsorption efficiency of the phospho (+) cells could be explained by the treatment of the dead phospho (+) cells with ethanol making the cell membranes permeable. Thus, P_3m_ could diffuse into the cells and phosphorylate the inner as well as the outer cell walls, allowing the whole structures (including the inner cell walls) of the phosphorylated cells to adsorb metal ions. Phosphorylating the yeast cells therefore strongly enhanced metal ion adsorption by the cells.

**Table 2:** Heavy metal ion removal (%) and adsorption capacity (in mmol/g DCW) of the phospho (+) and phospho (−) yeast cells.

		Cu^2+^	Zn^2+^	Pb^2+^	Cd^2+^
Phospho (+) cells	Removal [%]	34.2 ± 5.6	38.3 ± 6.2	94.7 ± 3.6	49.2 ± 8.8
	Adsorption capacity [mmol/g DCW]	1.08 ± 0.18	1.17 ± 0.19	0.91 ± 0.03	0.88 ± 0.16
Phospho (−) cells	Removal [%]	6.8 ± 3.3	8.7 ± 5.7	27.0 ± 9.6	10.6 ± 4.4
	Adsorption capacity [mmol/g DCW]	0.21 ± 0.10	0.26 ± 0.17	0.26 ± 0.09	0.19 ± 0.08

The metal ion concentration was 100 ppm, and the yeast cell concentration was 0.5 mg/mL.

Table 3 shows a summary of the adsorption capacities of metal ions using dead biomass of *S. cerevisiae*. The maximum adsorption capacities of Pb^2+^ and Cd^2+^ in the literatures were 1.31 and 0.77 mmol/g DCW, respectively^26,27^. These values were comparable with the values in this study. However, it seems that this adsorption occurred with specific combinations between the type of metal ion and specifically treated cells. In contrast, whereas the maximum Cu^2+^ and Zn^2+^ adsorption capacities in the previous studies were approximately 0.2 mmol/g DCW, the values in this study were significantly higher (~1.0 mmol/g DCW), which suggests that the phospho (+) cells proposed in this study have broad utility for various types of metal ions.

**Table 3:** Adsorption capacities of heavy metal ions using dead biomass of *S. cerevisiae*.

Source or form	Metal ion	Adsorption capacity [mmol/g DCW]	Reference
Phosphorylated dry baker’s yeast	Cu^2+^	1.08	This study
	Zn^2+^	1.17	
	Pb^2+^	0.91	
	Cd^2+^	0.88	
Whisky distillery spent wash lyophilized	Cu^2+^	0.09	Bustard and McHale^30^
	Zn^2+^	0.26	
	Pb^2+^	0.91	
Lab cultivated, then dried at 100 °C	Pb^2+^	1.31	Ozer and Ozer^26^
Lab cultivated, then dried at 45 °C	Cu^2+^	0.17	Machado *et al*.^25^
	Zn^2+^	0.16	
Inactive instant dry baker’s yeast	Cd^2+^	0.44	Stoica *et al*.^31^
Alkali-treated lyophilized yeast	Cr^2+^	0.10	De Rossi *et al*.^14^
Heat (121 °C)-treated lyophilized yeast	Cr^2+^	0.13	
Ethanol-treated waste baker’s yeast	Pb^2+^	0.08	Goksungur *et al*.^32^
	Cd^2+^	0.14	
Deactivated protonated yeast	Cd^2+^	0.77	Vasudevan *et al*.^27^

### Desorption of metal ions from the phosphorylated yeast cells

**Figure 4:**
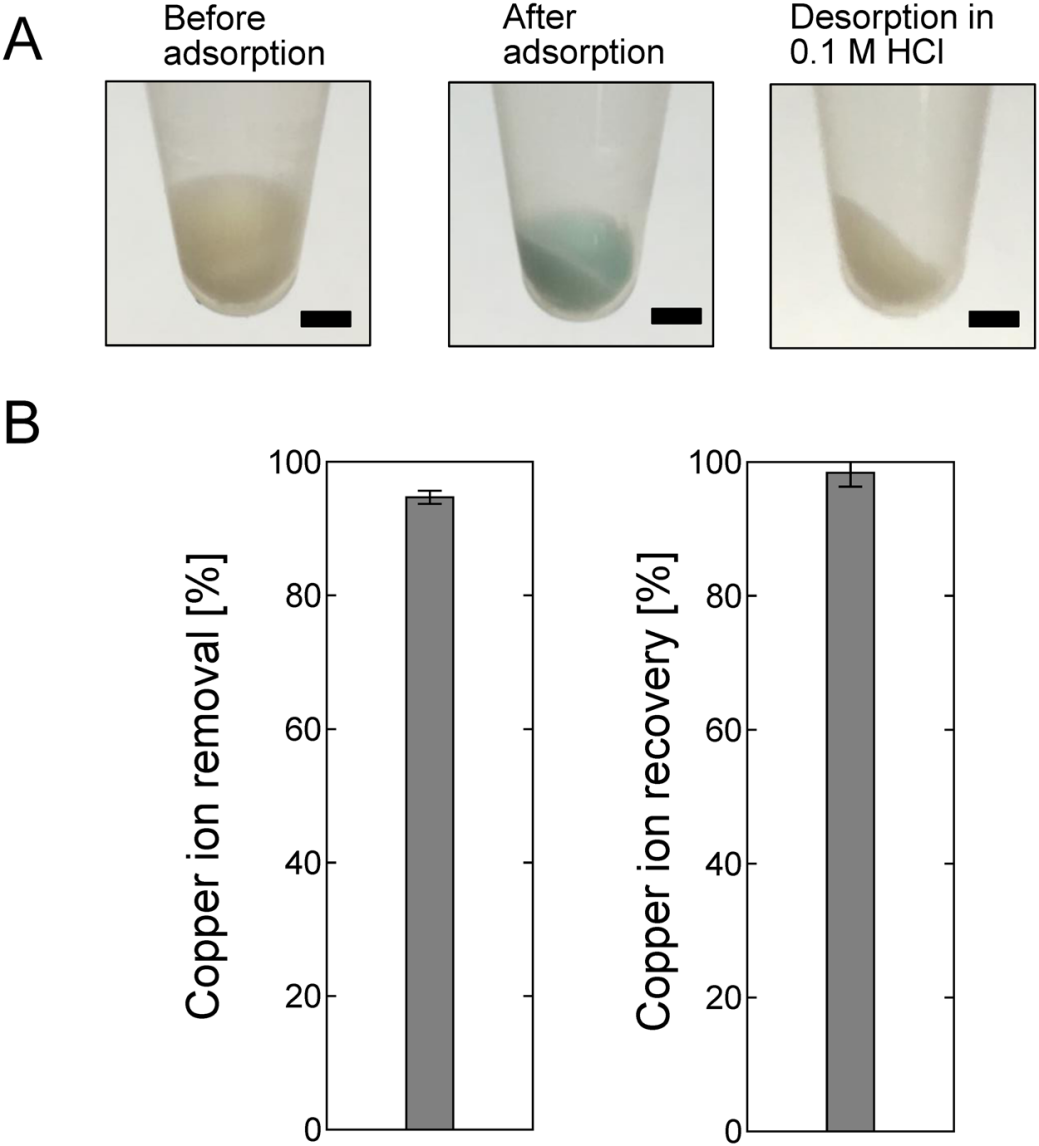
Desorption of adsorbed Cu^2+^ under acidic conditions. (**A**) Photographs of the phospho (+) yeast cells before and after Cu^2+^ had been adsorbed and after the Cu^2+^ had been desorbed by exposing the cells to 0.1 M HCl (each scale bar indicates 2 mm). The metal ion concentration was 100 ppm and the yeast cell concentration was 2.5 mg/mL. (**B**) (left) Cu^2+^ removal by phospho (+) yeast cells and (right) Cu^2+^ recovery in 0.1 M HCl.

The desorption of metal ions from the phosphorylated yeast cells is important in terms of recovering metals removed from solution by the cells. It has previously been found that the adsorption of metal ions onto phosphorylated cellulose paper was strongly influenced by the pH of the solution and that almost no adsorption occurred when the paper was treated with 0.1 M HCl^22^. We therefore attempted to desorb Cu^2+^ ions from the phosphorylated yeast cells using 0.1 M HCl. Photographs of the phospho (+) cells before and after adsorption of Cu^2+^ ions and after the ions had been desorbed using HCl are shown in Fig. 4A. The Cu^2+^ ion concentration in the test solution was 100 ppm, and the yeast cell concentration was 2.5 mg/mL. The phospho (+) cells were white before being exposed to Cu^2+^ ions and light blue when Cu^2+^ ions had been adsorbed. The cells with adsorbed Cu^2+^ ions turned white again when treated with HCl, suggesting that the Cu^2+^ ions were successfully desorbed into the 0.1 M HCl. The adsorption and desorption of Cu^2+^ ions were quantified by performing inductively coupled plasma (ICP) analyses. As shown in Fig. 4B, approximately 95% of the Cu^2+^ ions in solution became adsorbed to the phospho (+) cells, and approximately 98% of the Cu^2+^ ions adsorbed to the cells became desorbed when the cells were treated with 0.1 M HCl. We concluded that 98% of the Cu^2+^ ions adsorbed to phospho (+) cells could be recovered simply by treating the cells with HCl.

### Adsorption of rare earth ions onto the phosphorylated yeast cells

**Figure 5:**
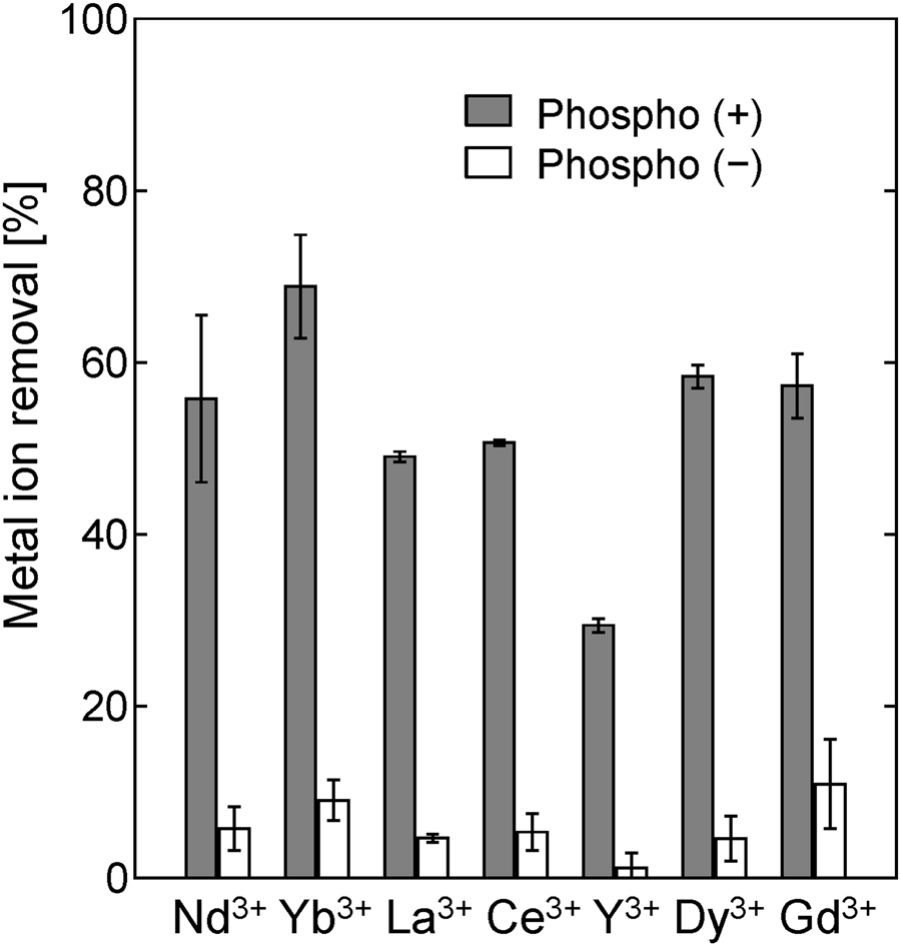
Percentages of Ce^3+^, Dy^3+^, Gd^3+^, La^3+^, Nd^3+^, Y^3+^, and Yb^3+^ removed from solution by the phospho (+) and phospho (−) yeast cells. The metal ion concentration was 100 ppm (Ce^3+^ 0.71 mM, Dy^3+^ 0.62 mM, Gd^3+^ 0.64 mM, La^3+^ 0.72 mM Nd^3+^ 0.69 mM, Y^3+^ 1.12 mM, and Yb^3+^ 0.58 mM), and the yeast cell concentration was 0.5 mg/mL.

The results presented in the previous section indicate that the phosphorylated yeast cells could adsorb heavy metals. However, only a few reports have described the biosorption of rare earth ions using *S. cerevisiae* biomass^28,29^. Therefore, we tested the ability of the phosphorylated yeast cells to adsorb seven rare earth ions (Ce^3+^, Dy^3+^, Gd^3+^, La^3+^, Nd^3+^, Y^3+^, and Yb^3+^). As shown in Fig. 5, the phospho (+) cells removed 50–70% of most of the rare earth ions from solution, whereas the phospho (−) cells removed <10%, which indicates that the phosphorylated yeast cells could be used to adsorb rare earth ions from solution. However, only ~30% of the Y^3+^ ions were removed by the phospho (+) cells, because Y has an atomic weight of 88.91, about half the atomic weights of the other rare earth elements (138.9–173.04). The phospho (+) and phospho (−) cell adsorption capacities for the rare earth ions were calculated. As shown in Table 4, the phospho (+) cell adsorption capacities were 0.7–0.8 mmol/g DCW, which were much higher than the phospho (−) cell adsorption capacities.

**Table 4:** Rare earth ion removal (%) and adsorption capacity (in mmol/g DCW) of the phospho (+) and phospho (−) yeast cells.

		Nd^3+^	Yb^3+^	La^3+^	Ce^3+^	Y^3+^	Dy^3+^	Gd^3+^
Phospho (+) cells	Removal [%]	55.8 ± 9.7	65.8 ± 6.0	49.0 ± 0.6	50.7 ± 0.3	29.4 ± 0.8	58.4 ± 1.3	57.3 ± 3.1
	Adsorption capacity [mmol/g DCW]	0.77 ± 0.13	0.76 ± 0.07	0.71 ± 0.01	0.72 ± 0.01	0.66 ± 0.02	0.72 ± 0.02	0.73 ± 0.05
Phospho (−) cells	Removal [%]	5.7 ± 2.5	9.1±2.3	4.6 ± 0.5	5.4 ± 2.1	1.2 ± 1.8	4.6 ± 2.6	10.9 ± 5.2
	Adsorption capacity [mmol/g DCW]	0.08 ± 0.04	0.10 ± 0.03	0.07 ± 0.01	0.08 ± 0.03	0.03 ± 0.04	0.06 ± 0.03	0.14 ± 0.07

The metal ion concentration was 100 ppm, and the yeast cell concentration was 0.5 mg/mL.

### Selective adsorption of rare earth ions from mixtures of rare earth ions and heavy metal ions

**Figure 6:**
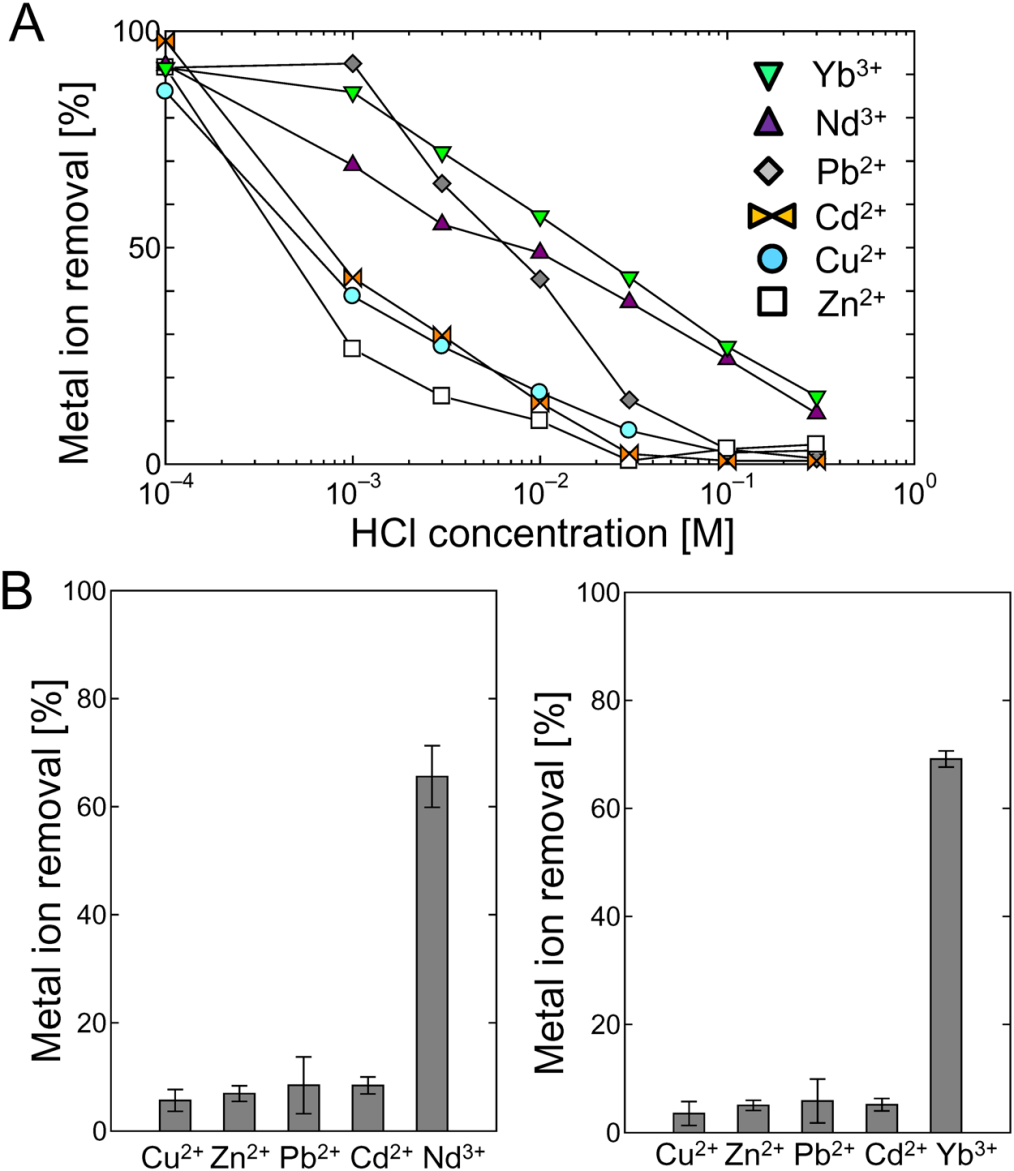
Selective adsorption of rare earth ions from solutions containing heavy metal and rare earth ions. (**A**) Influence of the HCl concentration on metal ion removal by the phospho (+) yeast cells. The metal ion concentration was 10 ppm and the yeast cell concentration was 0.2 mg/mL. (**B**) Simultaneous adsorption of Nd^3+^ or Yb^3+^ and the heavy metal ions Cd^2+^, Cu^2+^, Pb^2+^, and Zn^2+^. Each metal ion concentration was 10 ppm and the yeast cell concentration was 0.4 mg/mL.

As mentioned above, Cu^2+^ ions adsorbed to the phosphorylated yeast cells were desorbed by 0.1 M HCl. We therefore acquired profiles for the adsorption of heavy metal and rare earth ions onto the phospho (+) cells at various HCl concentration. The adsorption profiles for four heavy metal ions (Cd^2+^, Cu^2+^, Pb^2+^, and Zn^2+^) and two rare earth ions (Nd^3+^ and Yb^3+^) are shown in Fig. 6 A. The percentages of the heavy metals adsorbed decreased dramatically as the HCl concentration increased from 0.0001 to 0.001 M. The amount of Cu^2+^ ions removed at a HCl concentration of 0.001 M was approximately 40% of the amount removed at a HCl concentration of 0.0001 M. The amount of Cu^2+^ ions removed gradually decreased as the HCl concentration increased further, and no Cu^2+^ ions were removed at a HCl concentration of 0.1 M. The amounts of Cd^2+^ and Zn^2+^ ions removed followed similar trends to the amount of Cu^2+^ ions removed, decreasing markedly as the HCl concentration increased from 0.0001 to 0.001 M solution and approaching zero at a HCl concentration of 0.1 M. The amount of Pb^2+^ ions removed followed a different trend, not decreasing dramatically as the HCl concentration increased from 0.0001 to 0.001 M HCl and remaining relatively high compared with the amounts of the other heavy metal ions removed. The amounts of rare earth ions removed followed different trends to the amounts of heavy metals removed. Between 70% and 90% of the rare earth ions were removed at a HCl concentration of 0.001 M, and the percentage removed decreased slightly as the HCl concentration was increased from 0.001 to 0.01 M, but remained in the range 50%–60%. Rare earth ions were therefore adsorbed more strongly than heavy metal ions by the phosphorylated yeast cells, which would have been because the rare earth ions are trivalent whereas the heavy metal ions are divalent.

These results led us to adjust the HCl concentration to selectively adsorb rare earth ions from a mixture of rare earth ions and heavy metal ions. We exposed the phosphorylated yeast cells to solutions containing four types of heavy metal ions and one type of rare earth ion. The results of adsorption tests using solutions containing four heavy metal ions (Cd^2+^, Cu^2+^, Pb^2+^, and Zn^2+^) and Nd^3+^ or Yb^3+^ in 0.01 M HCl are shown in Fig. 6B. Each metal ion was used at a concentration of 10 ppm, and the phospho (+) cell concentration was 0.4 mg/mL. Interestingly, ~10% of each heavy metal ion was removed but ~70% of the Nd^3+^ was removed, which indicates that the Nd^3+^ was selectively adsorbed by the phosphorylated yeast cells. A relatively high percentage of the Pb^2+^ ions was adsorbed in 0.01 M HCl (Fig. 6A), but Nd^3+^ was still selectively adsorbed from mixtures including Pb^2+^. The adsorption of Yb^3+^ from a solution also containing heavy metal ions followed a similar trend, with ~70% of the Yb^3+^ being removed from the solution. This was a much higher percentage than the percentages of the heavy metal ions that were removed. These results suggest that various rare earth ions could be selectively adsorbed by the phospho (+) cells. These results demonstrate that the phosphorylated yeast cells preferentially adsorbed rare earth ions from a mixture of rare earth ions and heavy metals, with means that phosphorylated yeast cells may find uses in the metal recycling field.

In conclusion, P_3m_ was used to phosphorylate yeast cells. The P content of the phosphorylated yeast cells was ~1 mmol/g DCW, and the zeta potential was −45 mV, twice as high as the zeta potential of the non-phosphorylated yeast cells. The phosphorylated yeast cells adsorbed heavy metal ions, giving maximum heavy metal contents of ~1 mmol/g DCW, comparable with the highest contents found in previous studies using yeast biomass. The adsorbed metal ions were easily desorbed by HCl. The phosphorylated yeast cells preferentially adsorbed trivalent rare earth ions (Nd^3+^ and Yb^3+^) from mixtures of heavy metal and rare earth ions. The phosphorylated yeast cells have great potential for use as bioadsorbents.

## Methods

### Phosphorylation of yeast cells

Commercial dry baker’s yeast (Nisshin Seifun Group, Tokyo, Japan) was used throughout the study. Yeast cells were first washed five times with pure water and then fixed with 70% (v/v) ethanol for 2 h. The yeast cells were then phosphorylated using sodium cyclo-triphosphate hexahydrate, Na_3_P_3_O_9_·6H_2_O, following a method previously used to phosphorylate cellulose^22^. The yeast cells were phosphorylated using a 20% P_3m_ solution at 50 °C for 5 d. The solution pH gradually decreased as the reaction progressed; therefore 6 M sodium hydroxide_(aq)_ was added (with stirring) to keep the solution at pH 12. After the reaction, the phosphorylated yeast cells were washed with distilled water and lyophilised. The phosphorylated cells are called phospho (+) cells. Negative controls (called phospho (−) cells) were prepared following the same procedure but without adding P_3m_.

### Characteristics of the phosphorylated yeast cells

The P contents of the phosphorylated yeast cells were determined using the vanadomolybdate method at the Center for Organic Elemental Microanalysis at Kyoto University. The zeta potentials of the phosphorylated and non-phosphorylated dry baker’s yeast cells, suspended in pure water to give an optical density at 600 nm of 0.5, were determined using a Zetasizer Nano ZS instrument (Malvern Instruments, Malvern, UK).

### Bioadsorption of heavy metal and rare earth ions by the phosphorylated yeast cells

Stock solutions of the metals of interest were prepared using 1,000 mg/L (1,000 ppm) of Cd^2+^, Ce^3+^, Cu^2+^, Dy^3+^, Gd^3+^, La^3+^, Nd^3+^, Pb^2+^, Y^3+^, Yb^3+^, and Zn^2+^. The stock solutions were used to prepare standards at the different concentrations required. Standards at different concentrations were analysed using a Varian Vista MPX simultaneous ICP optical emission spectrometer (Agilent Technologies, Santa Clara, CA, USA).

Each adsorption test involved suspending yeast cells at a concentration of between 0.2 and 2.5 mg DCW/mL in 4 or 10 mL of a metal ion solution at a concentration of 10 or 100 ppm (prepared by diluting the appropriate stock solution with pure water) in a test tube. The test tube was shaken on a reciprocal shaker at 140 rpm at 30 °C for a specified time; the mixture was then centrifuged at 3,000 g for 10 min and then at 17,800 g for 5 min to remove the cells and debris. The adsorption tests using rare earth ions were performed using 0.0001 M HCl to allow the effects of phosphorylation to be clearly identified. The supernatant solutions after centrifugation were analysed by ICP optical emission spectrometry.

### Desorption of copper ions adsorbed to the phosphorylated yeast cells

Metal ions adsorbed to the phosphorylated yeast cells were desorbed by adding 0.1 M HCl and allowing the mixture to stand for 3 h. The supernatant was analyzed by ICP optical emission spectrometry and the copper ion recovery efficiency was calculated.

### Selective adsorption of rare earth ions from a mixture of heavy metal and rare earth ions

The heavy metal and rare earth ion adsorption profiles for the phospho (+) cells at various HCl concentration were determined in preliminary experiments. In these experiments, the metal ions were used at a concentration of 10 ppm and the yeast cells at a concentration of 0.2 mg/mL. The HCl concentrations were 0.00001–0.3 M. Selective adsorption of rare earth ions was studied by performing adsorption experiments using mixtures of four heavy metal ions (Cd^2+^, Cu^2+^, Pb^2+^, and Zn^2+^) and either Nd^3+^ or Yb^3+^ in 0.01 M HCl. In these experiments, the metal ions were used at a concentration of 10 ppm and the yeast cells at a concentration of 0.4 mg/mL. The test tubes containing the cells and test solutions were shaken on a reciprocal shaker at 140 rpm at 30 °C for 10 min. The mixtures were then centrifuged and the supernatants were analysed by ICP optical emission spectrometry.

### Statistical analysis

Each result is presented as the mean ± the standard deviation for more than three independent experiments except in Fig. 6A, for which *n* was 2.
